# Dr. Alonzo S. Hinkley

**Published:** 1898-04

**Authors:** 


Dr. Alonzo S. Hinkley, of Buffalo, died at the hospital of the Sisters of Charity, February 25, 1898, aged 75 years. Dr. Hinkley came to Buffalo in 1856, where he was engaged in the practice of homeopathic medicine until his recent illness. Besides a widow he leaves one daughter, Mrs. Frederick H. Ferguson, of Buffalo, and six sons, Arthur A., of New York, and Elmer B., George B., Willard E., Frank W. and Alonzo G. of this city.

